# Copy number deletion of PLA2G4A affects the susceptibility and clinical phenotypes of schizophrenia

**DOI:** 10.1038/s41537-024-00474-0

**Published:** 2024-05-30

**Authors:** Zibo Gao, Xinru Guo, Zhouyang Sun, Songyu Wu, Qianyi Wang, Qianlong Huang, Wei Bai, Changgui Kou

**Affiliations:** Department of Epidemiology and Biostatistics, School of Public Health, Jilin University, Changchun, Jilin China

**Keywords:** Epigenetics in the nervous system, Schizophrenia

## Abstract

Phospholipase A2(PLA2) superfamily is recognized as being involved in the pathogenesis of schizophrenia by affecting lipid homeostasis in cell membranes. We hypothesized that PLA2 gene copy number variation (CNV) may affect PLA2 enzyme expression and be associated with schizophrenia risk. This study indicated that in the discovery stage, an increased copy number of PLA2G6 and the deletion of PLA2G3, PLA2G4A, PLA2G4F and PLA2G12F was associated with increased risk of schizophrenia. CNV segments involving six PLA2 genes were detected in publicly available datasets, including two deletion segments specific to the PLA2G4A gene. The relationship between the deletion of PLA2G4A and susceptibility to schizophrenia was then reaffirmed in the validation group of 806 individuals. There was a significant correlation between PLA2G4A deletion and the symptoms of poverty of thought in male patients and erotomanic delusion in females. Furthermore, ELISA results demonstrate a significant decrease in peripheral blood cytosolic PLA2(cPLA2) levels in patients with the PLA2G4A deletion genotype compared to those with normal and copy number duplicate genotypes. These data suggest that the functional copy number deletion in the PLA2G4A gene is associated with the risk of schizophrenia and clinical phenotypes by reducing the expression of cPLA2, which may be an indicator of susceptibility to schizophrenia.

## Introduction

Schizophrenia is a serious psychiatric disorder^1^, characterized by shortened life expectancy, increased risk of suicide, and profound systemic symptoms^2,3^. The etiology of schizophrenia is complex, with 64–81% of schizophrenia traits attributed to genetic factors^3^. In recent years, scholars have gradually identified numerous genetic variations and susceptibility genes contributing to the genetics of schizophrenia through methods such as genetic typing and sequencing studies^4–6^. However, current research on the etiology of schizophrenia remains insufficient, and many aspects of how risk genes function and the extent of their risk have yet to be elucidated. Over the past decade, researchers have discovered that copy number variations (CNVs) are also significant heritable variations^7^. CNV is considered to be a genomic structural variant greater than 1 kb in length, which is characterized by copy number duplication or deletion in large segments of the genome compared to the reference genome^8,9^. Large-scale genetic studies have identified multiple copy number variants that contribute to different psychiatric disorders including schizophrenia, autism, and intellectual disability/developmental delay^7^. Among them, the major independent copy number variant loci considered to be strongly associated with the risk of schizophrenia include 1q21.1, 2p16.3 (NRXN1), 3q29, 7q11.23, 15q11-q13, 15q13.3, 16p13.11, 16p12.1, 16p11.2, 22q11.2^10–12^. However, due to the substantial heterogeneity and low repeatability of CNVs across different populations, and the challenge of obtaining statistical evidence for rare segments, as well as the potential for recurrent variations affecting multiple gene loci, CNV associated with schizophrenia susceptibility genes remains underexplored^13^.

The etiologic mechanisms of schizophrenia are complex and are influenced by genetic factors^14,15^, neurochemical changes^16,17^, structural changes in the brain^18,19^, and a variety of other high-risk factors^20,21^, and the etiologic mechanisms have yet to be elucidated. As far back as the 1990s, Horrobin’s group^22,23^ proposed the membrane phospholipid hypothesis of schizophrenia. Based on the scientific premise that normal neuronal phospholipid metabolism is important in the normal development of brain structures, pubertal regulation, and maintenance of neuronal function in adulthood, the hypothesis states that abnormal phospholipid metabolism in brain cells and red blood cells of patients with schizophrenia is characterized by abnormal metabolism of essential fatty acids (EFAs) such as arachidonic acid (AA) and docosahexaenoic acid (DHA). Using mass spectrometry (MS) and bioinformatics techniques, Li^24^ have discovered that the lipid homeostasis of cell membranes in schizophrenic patients is disrupted under pathological conditions, potentially linked to dysregulation of oxidative stress dysregulation. This lipid abnormality may lead to dysfunction in the central nervous system membrane function and neurotransmitter transmission in patients. Brain tissue is high in phospholipids, and PLA2 is a key link involved in membrane phospholipid metabolism. Here, we focus on the phospholipase A2 (PLA2) superfamily of genes, which primarily encode phospholipase A2 enzymes. These enzymes primarily use phosphatidylglycerol as a substrate and release free fatty acids and lysophospholipids by hydrolyzing their sn-2 position acyl bond^25,26^. Our preliminary research has also revealed that abnormalities in PLA2 gene SNPs, enzyme activity, and its concentration may represent a significant etiological clue for schizophrenia^27–30^. However, there is currently no research on CNVs in PLA2 genes and their relationship with schizophrenia.

Therefore, this study investigated the relationship between the copy number of PLA2 superfamily genes and schizophrenia in two phases. We combined whole-genome sequencing data with targeted detection data to identify potential key PLA2 CNVs. Subsequently, we expanded the sample size for further validation to examine whether selected CNVs of PLA2 genes are associated with susceptibility to schizophrenia or clinical phenotypes among patients in the Han Chinese population. Simultaneously, we explored the relationship between different genotypes and the clinical manifestations of patients, as well as the expression levels of PLA2 enzymes.

## Materials and methods

### Subjects

A total of 504 schizophrenia patients and 502 corresponding unrelated controls were enlisted for the study. The patients were specifically recruited from the Mental Health Institution of Sixth People’s Hospital in Jilin, China. The diagnosis of schizophrenia was confirmed by at least two experienced psychiatrists using the International Statistical Classification of Diseases and Related Health Problems, Tenth Revision (ICD-10). The clinical data of participants in this study, all indicate whether the phenotype under investigation exists in the study subjects, were collected through standardized assessment conducted by trained clinical physicians. Schizophrenic patients included in the study have no history of surgery, corticosteroids, procoagulant drugs, angiotensin-converting enzyme inhibitors, angiotensin II receptor antagonists, adrenergic agents, or any similar medications within 3 months prior to enrollment. Patients must be older than 18 years of age, and there should be no blood relationship between the cases. The controls were individuals undergoing health examinations at the First Hospital of Jilin University. Only unaffected individuals with no history of psychiatric disorder or familial predisposition were included in the control group. All study participants or their families provided informed consent. Furthermore, this research complied with the Declaration of Helsinki and received approval from the Ethics Committee of the School of Public Health, Jilin University (NO. 2020-04-06).

### CNV genotyping and validation

#### DNA extraction

The genomic DNA of case and control group were extracted from peripheral blood sample (0.6–1 mL) using blood genomic DNA extraction kit (DP348 and DP335, TIANGEN Biotech Co. Ltd., Beijing, China) according to the manufacturer’s instructions. DNA samples were stored at −80 °C. The concentration of DNA was determined using Epoch ultramicro microplate reader (BioTek Co. Ltd. America) before genotype detection.

#### CNVplex assay

A multiplex gene copy number quantitation method, CNVplex assay, was employed to genotype 9 CNV loci in the discovery stage in an independent population consisting of 100 cases and 100 controls. The method can simultaneously detect multiple CNVs in one reaction by using multiplex fluorescence PCR to amplify ligation products with four different dye-labeled universal primers^31^. The experiments were performed under the instruction of technologists from Genesky Diagnostics (Suzhou city, China)^32^. The sequences of the probes targeting these CNV loci are listed in Table S1. In brief, 4 μL of DNA sample was initially denatured at 98 °C for 5 min in a 10 μL reaction containing DNA lysis Buffer. Subsequently, add the ligation reaction premix to the denatured DNA sample, and perform ligation reaction under the action of ligase. Then, 1 μL Ligation Product was added in multi-fluorescent PCR reaction premix to amplify ligation products. The multiplexed fluorescent PCR reaction products were subjected to a 20-fold dilution before sequencing on the ABI 3730XL sequencer, followed by subsequent analysis of the raw data using GeneMapper v4.1 software (Applied Biosystems, USA).

#### CNV analysis in the GEO and SRA data set

To detect PLA2 family CNV, a group of DNA microarray data and a set of whole-genome sequence (WGS) data were obtained from the GEO database and the database NCBI SRA, respectively.

Firstly, a series of Affymetrix 6.0 array (Platform Accession No. GPL6801) CEL files were obtained from GEO database (GEO Database Accession No. GSE23201) which contained 245 schizophrenia cases and 490 controls^33^. The PennCNV software^34^ was utilized for the identification of CNV in this batch of data. The raw CEL files were initially transformed into raw intensity data through the PennCNV Affy workflow. Subsequently, the total signal intensity, represented by log R ratio (LRR), and the B allele frequency (BAF) were computed using the normalize affy geno cluster.pl program within the PennCNV-Affy package. And the scan_region.pl script file was used to annotation. Only samples meeting the criteria of LRR SD < 0.35 and BAF drift < 0.01 were considered for inclusion in this study. We limited our analysis to CNV regions that over 50 kb in size and overlapped more than 10 SNP probes.

In addition, we utilized raw data of schizophrenia patients and correspondent controls from the NCBI SRA database (NO. SRP000544). This project conducted whole-genome sequencing of 29 schizophrenia patients and 25 controls^35^. Raw whole-genome sequencing data were quality-checked using FastQC v.0.11.9 before being pre-processed with Trimmomatic v.0.39 to remove adapters and perform trimming to remove adapter sequences and low-quality bases. Control-FREEC(v.11.6)^36^ was used to detect genomic segments with CNVs under default parameters. And annotation was performed using ANNOVAR software. CNV segments 50 kb in size were deleted.

#### Screening for key PLA2 family gene

We searched for CNV segments related to the PLA2 superfamily among two genome-wide detected results, targeting the three main gene families: cytosolic PLA2 (cPLA2), secreted PLA2 (sPLA2), and calcium-independent PLA2 (iPLA2), comprising a total of 26 genes (i.e., iPLA2: PLA2G6, PNPLA1, PNPLA2, PNPLA3, PNPLA4, PNPLA5, PNPLA6, PNPLA7 and PNPLA8; sPLA2: PLA2G1B, PLA2G2A, PLA2G2C, PLA2G2D, PLA2G2E, PLA2G2F, PLA2G3, PLA2G5, PLA2G10, PLA2G12A and PLA2G12B; cPLA2: PLA2G4A, PLA2G4B, PLA2G4C, PLA2G4D, PLA2G4E and PLA2G4F).

The CNVplex detection results were compared with the results from two datasets. Following validation of frequency in the Database of Genomic Variants (DGV) database and determination of pathogenicity using AnnotSV 3.1.3 software, key PLA2 family genes associated with CNV were selected based on the following criteria: (1) The results of the targeted screening analyses and two genome-wide analyses showed that the CNV status of the selected genes was associated with schizophrenia; (2) The genes with a lower frequency of CNV in the DGV database than the cases screened in this study; (3) The selected genes were located in CNV regions that have been classified as “pathogenic” or “possibly pathogenic” by the ACMG guidelines.

#### qPCR assay

The PLA2G4A gene deletion was further genotyped in a larger population during replication stage 2 using the qPCR assay. The primers, listed in Supplementary Table S2, were designed using the NCBI Primer-BLAST tool (http://www.ncbi.nlm.nih.gov/tools/primer-blast/). Reactions were conducted on an ABI QuantStudio 3 Real‑Time PCR System (Applied Biosystems, Foster City, CA, USA) using SYBR Green reagents (Mei5 Biotechnology, Co., Ltd., Beijing, China) in total volumes of 10 μL which contained 5 μL 2 × M5 HiPer Realtime PCR Super mix with Low Rox, 250 nM of each primer, and 20 ng of genomic DNA. Amplification of the samples was carried out using the following cycling conditions: an initial denaturation step at 95 °C for 1 min, followed by 40 cycles of denaturation at 95 °C for 15 s, annealing at 60 °C for 15 s, and extension at 72 °C for 30 s. The analysis of results was performed utilizing QuantStudio Design & Analysis Software v1.5.1 (Applied Biosystems, Foster City, CA, USA) and subsequently subjected to further analysis employing the $\Delta\Delta$CT method. The GAPDH, SNCA, and POLR2A genes served as internal reference genes, with the geometric mean of their Ct values being utilized as the reference metric. The average of values from genomic DNA of two randomly selected control samples, which were determined to have a two-copy number in the results of the CNVplex assay, was considered the normal reference sample for $\Delta\Delta$CT calculations. Deletions were identified if the normalized absolute copy number value for a sample was below 1.2, while duplications were recognized when the value exceeded 2.8.

### CPLA2 determination

Each subject’s venous blood was collected in an anticoagulation tube containing EDTA and subsequently preserved at −80 °C. Anticoagulated whole blood was subjected to two cycles of freeze-thawing, followed by centrifugation at 3000 rpm for 20 min before collecting the supernatant for testing. The concentration of cPLA2 in whole blood was determined using an ELISA kit (MyBioSource Corp., USA). Briefly, take 50 μL of standard and pre-treated blood samples to be tested and add them to the well plate accompanying the kit. Subsequently, 100 μL of HRP-antibody was added to each well and incubated at 37 °C for 1 h. In the previous steps, no reagents were added to the blank wells. All wells were washed 4 times at the end of incubation. Subsequently, the reaction substrate was added and incubated at 37 °C for 15 min under light-avoidance conditions. After termination of the reaction, the OD value at 450 nm was read using microplate reader (Thermo Fisher Scientific Co. Ltd. America).

### Statistical analysis

Demographic characteristics distribution differences between cases and controls were assessed using the *χ*^2^ test for statistical analysis. The absolute copy number value of 2 copies was considered normal. Logistic regression analysis was employed to compute the associations between CNV genotypes and the risk of schizophrenia, with age and gender considered as covariates. And in gender-stratified analyses, logistic regression included age as a covariate. Furthermore, the *χ*^2^ test and Fisher’s exact test were used to analyze the effect of PLA2G4A CNV on schizophrenia clinical phenotypes and the differences in genotypes among different demographic characteristics. One-way ANOVA and LSD tests were used to compare the level of cPLA2 among different groups. All statistical analyses were conducted using SPSS 21.0 (IBM, Chicago, IL, USA), and statistical significance was determined by a two-sided *p* value < 0.05.

## Results

### Characteristics of the study population

In the discovery stage, we finally enrolled 99 schizophrenia cases (52 males, 47 females) and 100 controls (54 males, 46 females), with one schizophrenia patient excluded due to poor data quality. Additionally, 404 cases (244 males, 160 females) and 402 controls (216 males, 186 females) were recruited as a validation population. In the discovery phase, the age range for both the case and control groups was 18–59 years, and in the validation phase, it was 18–80 years for the case group and 18–82 years for the control group. No significant deviations were observed in the distributions of age and gender between cases and controls in both populations (*p* > 0.05). The distribution of demographic characteristics of the discovery and validation sets is outlined in Supplementary Table S3.

### CNV analysis of PLA2 gene in discovery stage

The main purpose of discovery stage was to identify the significant CNV loci of PLA2 family using multiple approaches. Firstly, we genotyped 9 PLA2 gene copy number in 100 schizophrenic patients and 100 matched controls by CNVplex technology. However, one patient with an abnormal result was removed. Thus, a total of 99 cases sequencing data were eventually included in the schizophrenia group. The result of CNVplex assay (see Table 1) indicated that the deletion of PLA2G3[odds ratio(OR) = 6.905, 95% confidence interval (95% CI) = 1.496–31.873; *p* = 0.013], PLA2G4A (OR = 8.357, 95% CI = 1.018–68.604; *p* = 0.048), PLA2G4F (OR = 6.261, 95% CI = 1.339–29.277; *p* = 0.020) and PLA2G12A (OR = 5.175, 95% CI = 1.094–24.481; *p* = 0.038), as well as the duplication of PLA2G6 (OR = 11.580, 95% CI = 3.352–40.006; *p* < 0.001) was significantly associated with increased risk of schizophrenia. In addition, PLA2G4D deletion were detected in 6 cases in the schizophrenia group, whereas the variant was not detected in any of the control samples; PLA2G12A duplication was detected in only 1 case in the schizophrenia group. Both genes may also serve as candidate copy number variant genes associated with susceptibility to schizophrenia.

**Table 1:** Frequency distribution of PLA2 genotypes from the CNVplex assay and their associations with schizophrenia risk.

Genes	CNV type	Schizophrenia [*n* (%)] (*n* = 99)	Control [*n* (%)] (*n* = 100)	OR (95% CI)	*p* value^a^
PLA2G3	Normal	86 (86.9)	92 (92.0)	1.000	
	Deletion	13 (13.1)	2 (2.0)	6.905 (1.496, 31.873)	**0.013**
	Duplication	0 (0.0)	6 (6.0)	–	–
PLA2G4A	Normal	88 (88.9)	97 (97.0)	1.000	
	Deletion	8 (8.1)	1 (1.0)	8.357 (1.018, 68.604)	**0.048**
	Duplication	3 (3.0)	2 (2.0)	1.683 (0.272, 10.409)	0.576
PLA2G4B	Normal	88 (88.9)	92 (92.0)	1.000	
	Deletion	3 (3.0)	1 (1.0)	2.802 (0.284, 27.666)	0.378
	Duplication	8 (8.1)	7 (7.0)	1.210 (0.418, 3.501)	0.725
PLA2G4C	Normal	96 (97.0)	96 (96.0)	1.000	
	Duplication	3 (3.0)	4 (4.0)	0.758 (0.164, 3.502)	0.722
PLA2G4D	Normal	89 (89.9)	98 (98.0)	1.000	
	Deletion	6 (6.1)	0 (0.0)	–	–
	Duplication	4 (4.0)	2 (2.0)	2.352 (0.417, 13.270)	0.333
PLA2G4E	Normal	91 (91.9)	97 (97.0)	1.000	
	Duplication	8 (8.1)	3 (3.0)	2.695 (0.687, 10.576)	0.155
PLA2G4F	Normal	83 (83.8)	93 (93.0)	1.000	
	Deletion	11 (11.1)	2 (2.0)	6.261 (1.339, 29.277)	**0.020**
	Duplication	5 (5.1)	5 (5.0)	1.159 (0.316, 4.245)	0.824
PLA2G6	Normal	71 (71.7)	95 (95.0)	1.000	
	Deletion	2 (2.0)	2 (2.0)	1.180 (0.158, 8.838)	0.872
	Duplication	26 (26.3)	3 (3.0)	11.580 (3.352, 40.006)	**<0.001**
PLA2G412A	Normal	88 (88.9)	98 (98.0)	1.000	
	Deletion	10 (10.1)	2 (2.0)	5.175 (1.094, 24.481)	0.038
	Duplication	1 (1.0)	0 (0.0)	–	–

–: not measured.

^a^Logistic regression analysis result with age and gender as covariables.

We detected 7175 and 17,881 effective CNV segments on autosomes in the GSE23201 and SRP000544 dataset, respectively. In the GSE23201 dataset, the PLA2G4A, PLA2G4B, PLA2G6, and PNPLA2 genes were covered by CNV fragments; in the SRP000544 dataset, CNV regions were detected in the PLA2G4A, PLA2G1B, PNPLA2, and PLA2G10 genes. Since duplications in the PLA2G10 gene were detected in both the case and control groups in the SRP000544 dataset, and the difference was not statistically significant (Fisher’s exact test *p* = 0.492), no subsequent analyses were performed. The other 5 genes were detected in 8 schizophrenia patients and 2 controls respectively. The details of the PLA2 CNVs were shown in Supplementary Material Table S4.

**Fig. 1:**
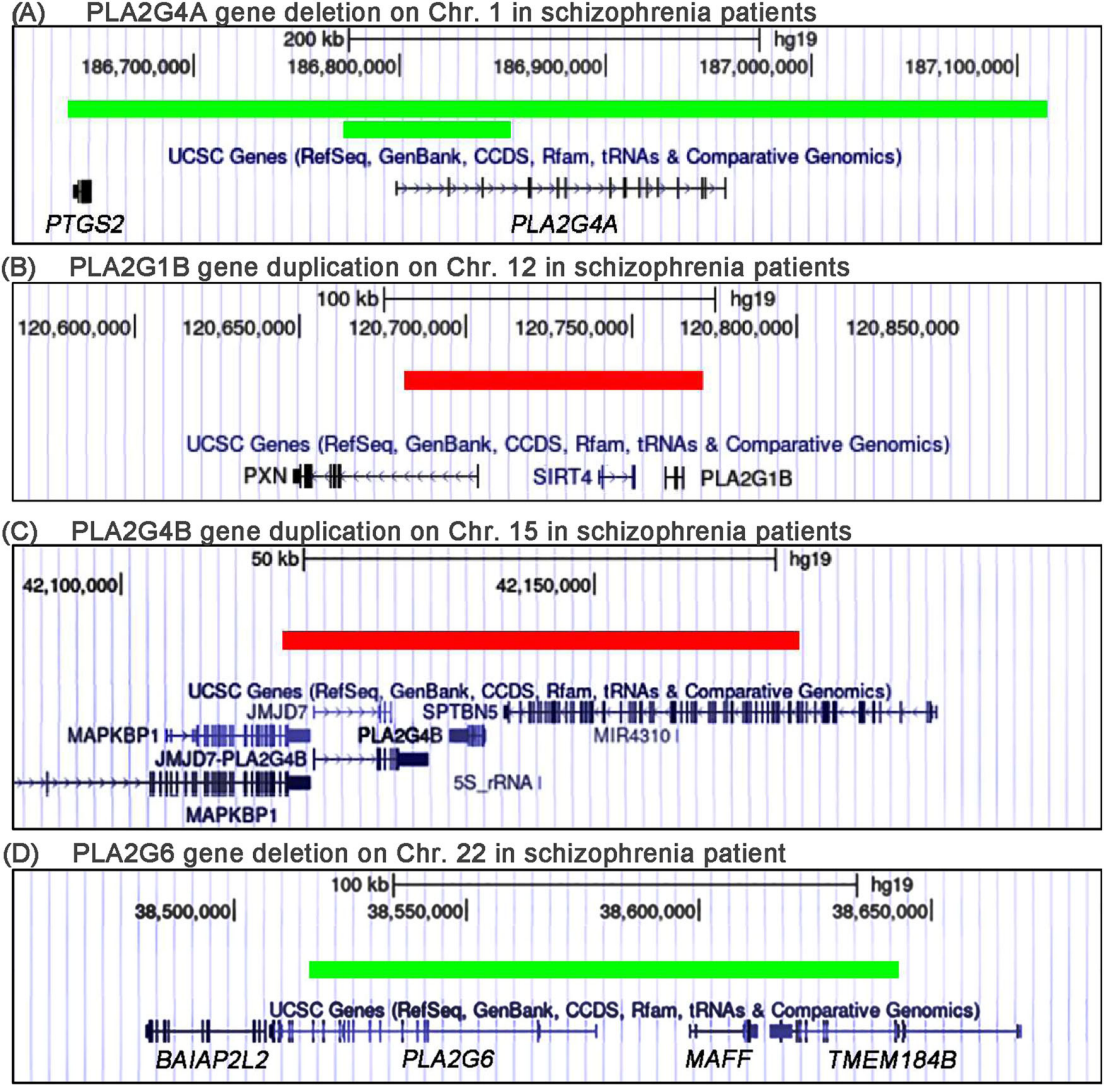
Five CNVs carrying PLA2 genes detected in open datasets. In this schematic representation, green means deletion, red equals duplication. **A** Two schizophrenia patients were identified to carry intragenic deletions of the PLA2G4A gene. **B** Duplication of the PLA2G1B gene was found in one schizophrenia patient. **C** Complete duplication of the PLA2G4B gene was found in one schizophrenia patient. **D** Partial deletion of the PLA2G6 gene was found in one patient.

Among the PLA2 CNVs, PLA2G4A deletion was repeated in the patient group (Fig. 1A). One schizophrenia patient from the SRA dataset was identified to carry complete deletion of PLA2G4A gene, which was classified as “pathogenic”. Another patient, from the GEO dataset, was identified to carry partial deletion of PLA2G4A gene spanning exons 1–3 of the PLA2G4A gene, which truncated the PLA2G4A coding sequence in the 5’-partial of the gene. And this CNV of PLA2G4Agene was classified as “points likely pathogenic”.

PLA2G1B duplication, PLA2G4B duplication and PLA2G6 deletion were also found in the disease (Fig. 1B–D). Furthermore, PNPLA2 gene region CNVs were detected in 5 cases (Supplementary Material Table S4). However, the PLA2G4B, PLA2G1B and PNPLA2 gene may have less priority, as they were embedded in CNVs with many flanking genes. Additionally, the CNVplex results indicated that PLA2G4B gene CNVs and the PLA2G6 gene deletion showed no association with schizophrenia.

Finally, by searching the database of DGV, we found one Gold Standard CNV of PLA2G4A partial deletion located on chr1:186870482–186871018(GRCh38) (Supplementary Fig. S1). The frequency of this CNV segment in the normal population is only 3.13% (27/863), significantly lower than the proportion of CNVs in the PLA2G4A gene in the population of cases in this screening stage 8.08% (8/99). This suggested that copy number variants in PLA2G4A may be associated with susceptibility to schizophrenia. Therefore, we selected the PLA2G4A as the final PLA2 gene to validate in stage 2.

### Validation in the qPCR

After comparing the deletion position of the PLA2G4A gene detected in the two sequencing datasets and DGV database, we designed specific primers in exon 3 of the PLA2G4A gene for the second stage of qPCR verification. Table 2 revealed significant differences between schizophrenia patients and controls, evident in the deletion of PLA2G4A (OR = 9.060, 95% CI = 3.820–21.492, *p* < 0.001). We then merged these two stages to increase the study power, considering their homogeneity (Supplementary Table S3). And the result suggested that the deletion genotype has an 8.98-fold increased risk of schizophrenia compared with 2-copy (OR = 8.981, 95% CI = 4.041–19.956; *p* < 0.001) (Table 3). Moreover, the analysis results in different genders indicate that the deletion of the PLA2G4A gene significantly increased the risk of schizophrenia in both males and females (Supplementary Table S5).

**Table 2:** The association between PLA2G4A genotypes and schizophrenia risk in validation stage.

Type	Patients (*n*, %)	Control (*n*, %)	OR (95% CI)	*p* value^a^
Normal	331 (81.9)	364 (90.5)	1.000	
Deletion	48 (11.9)	6 (1.5)	9.060 (3.820, 21.492)	<0.001
Duplication	25 (6.2)	32 (8.0)	0.866 (0.502, 1.495)	0.606

^a^Logistic regression analysis result with age and gender as covariables.

**Table 3:** The association between PLA2G4A genotypes and schizophrenia risk in overall population.

Type	Patients (*n*, %)	Control (*n*, %)	OR (95% CI)	*p* value^a^
Normal	419 (83.3)	461 (91.8)	1.000	
Deletion	56 (11.1)	7 (1.4)	8.981 (4.041, 19.956)	<0.001
Duplication	28 (5.6)	34 (6.8)	0.915 (0.545, 1.537)	0.736

^a^Logistic regression analysis result with age and gender as covariables.

### Association between the genotypes of PLA2G4A and the level of cPLA2

**Fig. 2:**
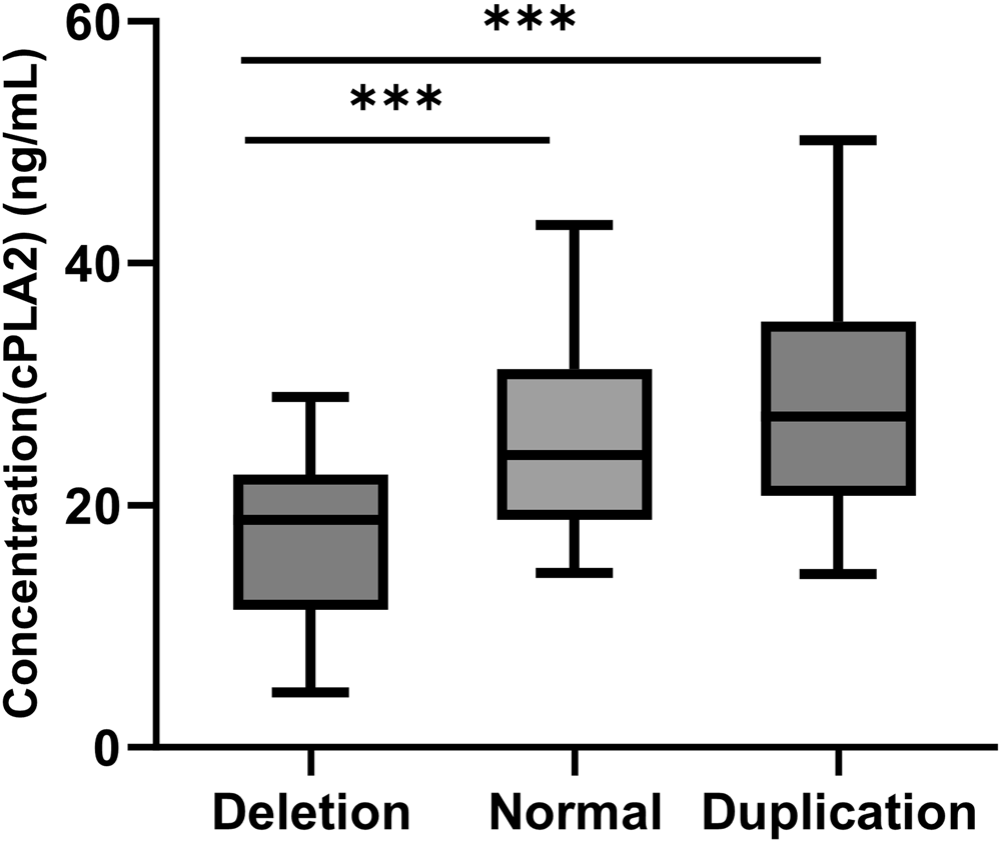
Peripheral blood cPLA2 levels in patients with different copy numbers of PLA2G4A gene. (***p < 0.001).

We measured the cPLA2 levels in the peripheral venous blood of the patients with different copy number genotypes by using the ELISA kits in randomly selected 30 patients with PLA2G4A deletion, 20 patients with duplicated PLA2G4A and 30 patients with normal genotype. As shown in Fig. 2, the mean cPLA2 concentration was 16.80 ± 7.15 ng/mL in patients with PLA2G4A deletion, 25.21 ± 7.93 ng/mL in patients with normal copy number, and 28.95 ± 10.26 ng/mL in patients with duplication. The ANOVA test showed that the overall difference in the distribution of cPLA2 concentrations among the three groups was statistically significant (*F* = 14.56, *p* < 0.001). The results of LSD showed that the cPLA2 concentration of patients with PLA2G4A deletion was significantly lower than that of those with normal (*p* < 0.001) and duplication (*p* < 0.001), and the difference between the normal copy number group and copy number duplicate group was not statistically significant.

### Association between the deletion of PLA2G4A and schizophrenia clinical phenotypes

We employed Chi-squared test and Fisher’s exact test to examine the associations between the genotype of PLA2G4A CNV and 29 clinical schizophrenia phenotypes (Supplementary Tables S6–S8). The results showed a significant correlation between PLA2G4A deletion and three symptoms in the overall patients: erotomanic delusion (*p* = 0.027), illogic of thinking (*p* = 0.048) and poverty of thought (*p* = 0.014). For male patients, there was a significant difference in poverty of thought (*p* = 0.009) among different genotypes. Among female patients, there were significant differences in the symptoms of erotomanic delusion (*p* = 0.034) between schizophrenia cases with PLA2G4A deletion CNV and those with other genotypes. The deletion of PLA24A gene may increase the risk of developing erotomanic delusion symptoms in female schizophrenia patients, while decreasing the risk of experiencing poverty of thought symptoms in male. These results indicate that the deletion of PLA2G4A gene is associated with the clinical schizophrenia phenotypes.

## Discussion

We explored the association between the copy number variation of PLA2 superfamily gene and schizophrenia in the Chinese Han population. To the best of our knowledge, this is the first study to investigate the association between the copy number variation of the PLA2 superfamily gene and susceptibility to schizophrenia, as well as its clinical phenotypes. We screened and validated that the deletion of the PLA2G4A gene from the PLA2 superfamily was significantly associated with susceptibility to schizophrenia. And we found that this variation may increase the risk of the disease by leading to a decrease in cPLA2 levels. Additionally, this study suggested that PLA2G4A gene deletion is significantly associated with erotomanic delusion, illogic of thought, and poverty of thought in the overall population, as well as poverty of thought in males and the erotomanic delusion phenotype in females.

PLA2G4A gene is located in 1q31.1, with a total length of 160Kb, contains 18 exons, and mainly encodes cPLA2α isoform functional protein^37^. Single nucleotide polymorphisms (SNPs) in the PLA2G4A have been demonstrated in several studies to potentially significantly correlate with the susceptibility to schizophrenia and its clinical symptoms^27,38–40^. Anna et al.^41^ detected a 2613Kb duplication segment in a schizophrenia patient, covering only the PLA2G4A gene, in an independent Munich cohort comprising 298 schizophrenia patients and 713 healthy controls. Additionally, a harmful novel variant SNV was detected in the PLA2G4A gene in one autistic patient in a genetic study^42^. These findings suggest that various types of mutations in the PLA2G4A gene may be closely associated with the mechanism underlying psychiatric disorders such as schizophrenia.

The BanI polymorphism located in the 5’UTR region of the PLA2G4A gene has undergone repeated validation, demonstrating a substantial correlation with the susceptibility to schizophrenia and clinical characteristics^38,43,44^. Among these characteristics, it may predominantly influence the negative symptoms in patients and the erotomanic delusion symptom in female individuals with schizophrenia^38,45^. In this study, we also observed similar findings. However, the association between illogic of thought and PLA2G4A deletion in this study shows inconsistencies between the analysis of the overall population and gender subgroups. This result may arise from minimal distribution differences of illogic of thought symptoms among patients with different types of PLA2G4A copy numbers, while gender factors exert a significant influence on the clinical phenotype association of schizophrenia. It is widely recognized in current research that schizophrenia tends to exhibit distinct patterns in different gender groups. Male patients with schizophrenia often display more noticeable negative symptoms compared to females, while women usually show more affective symptoms^46^. Ponizovsky observed a marked distinction in the phospholipid metabolism status of red blood cells between individuals exhibiting negative symptoms and those manifesting positive symptoms in schizophrenia. They identified that elevated levels of sphingolipids may constitute one of the contributing factors in the development of negative syndrome in schizophrenia^47^. Additionally, PLA2 enzyme activity has been found to be influenced by estrogen stimulation^48^, and the expression levels of the PLA2G4A gene, in turn, regulate the expression levels of estrogen receptor^49^. Therefore, the observed correlation between the CNV of PLA2G4A gene and different symptoms in various gender populations in this study is potentially regulated and controlled by sex hormones.

PLA2 enzymes, which primarily include the sPLA2, cPLA2, and iPLA2 three major classes, possess the ability to hydrolyze glycerophospholipids at the SN-2 position, resulting in the release of fatty acids and lysophospholipids. They play crucial roles not only in the generation of lipid mediators but also in processes such as phospholipid membrane remodeling, wound healing, cell proliferation and survival, as well as neuronal function^50^. The PLA2G4A gene primarily encodes cPLA2α (group IVA PLA2). AA, a key breakdown product of cPLA2, is considered a neuromodulator in the central nervous system^51^. The marked specificity of cPLA2α for AA at the sn-2 position of its phospholipid substrates suggests a potential crucial role of cPLA2 in neuronal plasticity^52,53^. This study suggests that copy number deletion of the PLA2G4A gene may increase the risk of schizophrenia by reducing the expression levels of cPLA2 enzyme. However, several studies indicate that levels of cPLA2 or its activity in individuals with schizophrenia may be higher than in healthy controls^54,55^, although this association remains controversial. Ross’ research revealed a significant decrease in calcium-dependent PLA2 activity in the post-mortem temporal cortex of schizophrenic patients compared to the control group, despite a 45% increase in iPLA2 in the patients’ brains^56^. Furthermore, a meta-analysis found that although there was a significant positive correlation between iPLA2 concentration and susceptibility to schizophrenia and its related niacin response, the levels of cPLA2 might not exhibit a significant association with susceptibility to schizophrenia^57^. The results of this study may offer a fresh insight into the contentious relationship between susceptibility to schizophrenia and the expression of cPLA2 enzyme.

Interestingly, in this study, deletions in the 5’ end exons 1–3 of the PLA2G4A gene were repeatedly detected in both sequencing datasets. The detection rate was significantly higher in the schizophrenia patient population compared to the healthy control group and the general population prevalence of exon 3 deletions in the DGV database. Combining previous research^52^ with bioinformatic analysis of protein structure (http://swissmodel.expasy.org; https://www.uniprot.org), we found that exons 1–3 may be involved in the phospholipid-binding function and the calcium-dependent membrane-targeting module. This suggests that the 5’ end of the gene may have a crucial impact on the expression and functionality of the PLA2G4A gene.

We identified a copy number variation segment in a schizophrenia patient involving a concurrent deletion of the PTGS2 and PLA2G4A genes. Both genes are located at the chromosomal position 1q25, approximately 149 Kb apart, and share a common promoter, forming a “head-to-head” structure. The PTGS2 gene is known to primarily encode the COX-2 enzyme, which plays a crucial role in catalyzing the rate-limiting step of prostaglandin formation from arachidonic acid^58^. These two genes may play a potential synergistic role in susceptibility to schizophrenia^59,60^ although this viewpoint remains subject to some controversy^61^. However, this study only designed primers for the PLA2G4A gene without performing breakpoint detection for CNV segments, which is a limitation as it may involve simultaneous variations in multiple genes. In addition, this study did not investigate the joint effects of environmental and genetic factors in schizophrenia patients, as well as the genetic pleiotropy of CNVs. Moreover, considering the observed differences in the detection rate of PLA2G4A CNV in the Northern Han Chinese population compared to data in the reference public databases, it is evident that there is significant heterogeneity in this CNV within the population. And we found that the odds ratio (OR) values and 95% confidence intervals for detecting PLA2G4A gene copy number deletion variants associated with disease in the two-stage population samples of this study had a wide range. Combined with the distribution of variant frequency data, this phenomenon may be due to the insufficiently large total sample size and the low frequency of variants in the control group. Moreover, although it has been suggested that the PLA2 superfamily genes influence the pathogenesis of schizophrenia by participating in cell membrane lipid homeostasis, the present study could not yet conclude that the pathomechanism of the association between deletion of the PLA2G4A gene and the risk of schizophrenia is related to cell membrane lipid disorders due to the lack of relevant experimental evidence. Therefore, in future studies, further validation in larger population samples is necessary to confirm the association between this variation and susceptibility to schizophrenia. Additionally, a more in-depth exploration of the potential mechanisms through which this variation may impact the onset and development of the disease is warranted, for example, the effect on cell membrane lipid homeostasis. Secondly, in samples detected with CNVs, further use of sequencing technology or qPCR methods should be employed to precisely detect the range of variations, while incorporating environmental factors into the analysis for comprehensive analysis. Finally, the detailed exploration and validation of how the deletion of the PLA2G4A gene copy number influences the risk of schizophrenia should be carried out using cellular or animal models, to establish a foundation for genetic variant screening and targeted therapies for schizophrenia.

In summary, this study suggested that the functional copy number deletion in the PLA2G4A gene could affect the risk of schizophrenia and clinical phenotypes by reducing the expression of cytosolic PLA2, which may be an indicator of susceptibility to schizophrenia.

### Supplementary information

Supplementary information

## Data Availability

Publicly available datasets which were analyzed in this study could be found in the NCBI SRA database (https://www.ncbi.nlm.nih.gov/sra, No. SRP000544) and GEO database (https://www.ncbi.nlm.nih.gov/geo/, No. GSE23201).
