# Supplementary material for: Copy number deletion of PLA2G4A affects the susceptibility and clinical phenotypes of schizophrenia
Source: Schizophrenia (Heidelb). 2024 May 30;10(1):55. doi: 10.1038/s41537-024-00474-0 (PMC11139948; doi:10.1038/s41537-024-00474-0)
Supplement: Supplementary file 1 — Supplementary information [file 41537_2024_474_MOESM1_ESM.pdf]

Supplementary Information

A list of supporting information:

Table S1 Probes used for CNVplex assays

Table S2 Primers used for qPCR assays

Table S3 Distribution of demographic characteristics for cases and controls included in the study

Table S4 CNVs carrying PLA2 genes detected in open datasets

Table S5 The association between PLA2G4A genotypes and schizophrenia risk in different genders

Table S6 Association between the deletion of PLA2G4A and schizophrenia clinical phenotypes in overall population

Table S7 Association between the deletion of PLA2G4A and schizophrenia clinical phenotypes in male

Table S8 Association between the deletion of PLA2G4A and schizophrenia clinical phenotypes in female

Figure S1 Schematic of the identified DGV Gold Standard CNV of PLA2G4A in Chr.1q31.1

Table S1 Probes used for CNVplex assays

Loci/genes (GRCh38)	Probes	5'Primer sequences	3'Primer sequences	Ligation product size (bp)
**For target Loci**				
PLA2G12A	1	TGCCTTGTCTCACTGGAACAGC	TGCCACCAATTCAAGTTGGAAG	157
PLA2G12A	2	GTCAAGGTCTGCCTTGCATCTG	TCACTCACACCTCCAGGATCTCC	149
PLA2G3	1	CTGAAAGCCCTCTCCAACACCC	ACCCCAATCCTGGATTCAGACA	120
PLA2G3	2	CTGAACCCCCATTCTGCACTG	GCCCAGTCCAGTCAGACAAAGC	103
PLA2G4A	1	GTCCGGAGCTGAAAAAGGATCC	TGACTGAAAGCTAGAGGCATTGAGG	153
PLA2G4A	2	GCAGTTTCTGATGCTGAGGCAG	TTTGCAATCCCATGACAACTGG	125
PLA2G4B	1	CCCTGGCTCTGGGTTAGAAAGC	TGACAGTCCTTGATCCTGTGGC	128
PLA2G4B	2	GCTGCTGCACCTGACACATTACA	ATGTCTGCAACAACCAGGAGCA	128
PLA2G4C	1	TGGACATGATGCTTCTGATTGTCA	GACACTCATGCTCCAGCTCCAG	150
PLA2G4C	2	CTGCTTGGTTGCTCCAGCTTTT	TCAGCTGTTCCAGAGCTCACCC	129
PLA2G4D	1	CAGTCCCTGAGCCAAGGTCTGT	CCCGAAGCTCTCACTACAACCC	132
PLA2G4D	2	CCAGCCCTTGCCAAGATGCT	GGCTCTCTGGCTGAGTGGCA	99
PLA2G4E	1	GCTGCCTTCGCCTTACGATAAA	CAAGTCCTGTCTGTTGCTTGGC	132
PLA2G4E	2	GTTTGTGGGCTCTGTGGGACAA	ACACATTAGTTCCCAGGCCAGG	133
PLA2G4F	1	GAACGGGATCTCCTCTTCACGA	ATCTGAGGGCTGCCCACACTAG	136
PLA2G4F	2	CTCCTCTGGTTGCACAAACTGC	TGGCTCAGGTGTGACAGGCA	103
PLA2G6	1	AGCAGCTGGATGAGCTTCTGGA	ACTCCTCGCGGTGCTCATAGAT	136
PLA2G6	2	CACCTCGGCTTACTCAGCACTTCT	ACTTCCTCCTACTTCCGCAAACAA	153
**For internal reference control**				
chr3:86978226-86978269	-	TTCCCTGTTACATAGTGCTGGGC	AGCCTCTGGGGGCCTCACA	95
chr9:72686936-72686983	-	GCCACTGGGGCTTTAGGAATGGT	ACTTCGCAGCTCAGTGCAGCC	108
chr4:55098216-55098265	-	CAGGCATAACAAACAGCTCAATTCCC	ACGCATGCAGCCCTCTCCA	124

chr6:70150672-70150724	-	GCTGGGGCCTTTTTTCAGCAT	CCTGGAGCCCTGAACAAGTACAAATG	141
chr8:47523679-47523720	-	GTGCCGGAGAAGATGATTCATGAC	TCTAGCCCTGCTTTTTCTCTCCCG	92
chr7:48045600-48045646	-	GCAAGTAGGATTGCAAAGGAGGAGG	TGAATGGCCAAGTCCTTTTGGAG	105
chr12:40827884-40827933	-	CAGCGCTCATTCTTTAGTCTCCGA	ACAGAATGACAGGGTTTGTGAAGTCG	121
chr17:27905628-27905679	-	TTCATCTGGATCCATGACGATGG	ACAAGTAGCCTGTCTTCAGTTCCCCTC	138
chr10:30830727-30830771	-	GCCACCCTCCAGTAGCCTTTTCCT	GGTGGTTCTGCATTGTACCTGAGAAA	95
chr3:87991398-87991445	-	TCACACAGAGTCACCCTCCTTCTGA	AGTGCTTTGCTTATAGCAGCCTGAACC	108
chr20:37237724-37237773	-	GGCTTCTCAGTTCTAAGCCTCACAAATC	CTTTCTACCTCCCACAGTGTTTGGG	124
chr9:37746606-37746659	-	GGGATGTGGTGTACACCTACCATCAGTT	TATAGAGGCTGCTAAATCGACCTGCG	141

Table S2 Primers used for qPCR assays

Target genes	Primers	Primer sequences (5'→3')	Ligation product size (bp)
PLA2G4A	Forward	TGGAGCACCAGTATTCCCACA	110
	Reverse	GGCACTTACGCATGTCACCAA	
**For internal reference control**			
GAPDH	Forward	TCAAGAAGGTGGTGAAGCAGG	111
	Reverse	AGGTGGAGGAGTGGGTGTCG	
SNCA	Forward	GCTGAGAAGACCAAAGAGCAAGTG	72
	Reverse	CTGGGCTACTGCTGTCACACC	
POLR2A	Forward	AGCAGAACGTCGAGGGCAAG	70
	Reverse	TCCTTGATGAAGTGAGGCAGAGTC	

Table S3 Distribution of demographic characteristics for cases and controls included in the study

		Schizophrenia	Control	χ^2	*P* value
Discovery stage (schizophrenia/control=99/100)					
Age(years)	0~20	8	4	2.460	0.483
	21~30	39	40		
	31~40	30	27		
	41~	22	29		
	Mean age (Mean±SD)	33.12±10.91	35.12±10.84		
Gender	Male	52	54	0.043	0.835
	Female	47	46		
Replication stage (schizophrenia/control=404/402)					
Age(years)	0~20	55	47	0.819	0.845
	21~30	143	141		
	31~40	123	126		
	41~	83	88		
	Mean age (Mean±SD)	32.81±11.73	33.67±12.21		
Gender	Male	244	216	3.653	0.056
	Female	160	186		
Overall population (schizophrenia/control=503/502)					
Age(years)	0~20	63	51	1.914	0.591
	21~30	182	181		
	31~40	153	153		
	41~	105	117		
	Mean age (Mean±SD)	32.87±11.57	33.96±11.95		
Gender	Male	296	270	2.617	0.106
	Female	207	232		

Table S4 PLA2 genes carrying CNVs detected in two open datasets

Dataset	Group	Copy number	Chromosomal region (hg19)	Size (bp)	PLA2 gene	Other genes	ACMG classification
GSE23201	Schizophrenia	1	chr1:186762025-186851483	89458	PLA2G4A	-	Points likely pathogenic
SRP000544	Schizophrenia	1	chr1:186634000-187115000	481000	PLA2G4A	PTGS2	Pathogenic
GSE23201	Schizophrenia	3	chr15:42117011-42171462	54451	PLA2G4B	5 others	Pathogenic
GSE23201	Schizophrenia	1	chr22:38520887-38646900	126013	PLA2G6	MAFF, TMEM184B	Pathogenic
SRP000544	Schizophrenia	3	chr12:120682000-120767000	85000	PLA2G1B	PXN, SIRT4	Points variant of uncertain significance
SRP000544	Schizophrenia	3	chr11:704000-829000	125000	PNPLA2	10 others	Points variant of uncertain significance
GSE23201	Schizophrenia	3	chr11:354390-2021199	1666810	PNPLA2	72 others	Pathogenic
GSE23201	Schizophrenia	1	chr11:742388-912466	170079	PNPLA2	13 others	Pathogenic
GSE23201	Control	3	chr11:699861-901992	202132	PNPLA2	14 others	Points variant of uncertain significance
GSE23201	Control	1	chr11:786554-923581	137028	PNPLA2	10 others	Pathogenic

Table S5 The association between PLA2G4A genotypes and schizophrenia risk in different genders

Type	Patients (n, %)	Control (n, %)	OR (95%CI)	*P* value[a]
Male				
Normal	254 (85.8)	248 (91.8)		
Deletion	26 (8.8)	4 (1.5)	6.369 (2.191, 18.519)	<0.001
Duplication	16 (5.4)	18 (6.7)	0.882 (0.439, 1.773)	0.725
Female				
Normal	165 (79.7)	213 (91.8)		
Deletion	30 (14.5)	3 (1.3)	12.532 (3.754, 41.837)	<0.001
Duplication	12 (5.8)	16 (6.9)	0.944 (0.433, 2.055)	0.884

[a]: Logistical regression analysis result with age as covariable

Table S6 Association between the deletion of PLA2G4A and schizophrenia clinical phenotypes in overall population

Symptoms	PLA2G4A CNV type		χ^2	*P* value
	**Deletion [n (%)] (N=56)**	**Non-deletion [n (%)] (N=447)**		
Auditory hallucinations	34 (60.7)	301 (67.3)	0.981	0.322
Olfactory hallucinations	0 (0.0)	4 (0.9)	-	1.000 [a]
Visual hallucinations	1 (1.8)	0 (2.2)	0.000	1.000
Visceral hallucination	0 (0.0)	3 (0.7)	-	1.000 [a]
Psychosensory disturbance	1 (1.8)	7 (1.6)	-	1.000 [a]
Delusion of reference	46 (82.1)	339 (75.8)	1.101	0.294
Delusion of persecutory	41 (73.2)	314 (70.2)	0.211	0.646
Delusion of physical influence	4 (7.1)	40 (8.9)	0.040	0.841
Delusion of jealousy	11 (19.6)	62 (13.9)	1.337	0.248
Delusion of sin	1 (1.8)	5 (1.1)	-	0.509 [a]
Delusion of grandeur	1 (1.8)	27 (6.0)	1.000	0.317
Nihilistic delusion	0 (0.0)	3 (0.7)	-	1.000 [a]
Erotomanic delusion	4 (7.1)	7 (1.6)	4.863	**0.027**
Catatatonia	1 (1.8)	5 (1.1)	-	0.509 [a]
Bizarre behavior	25 (44.6)	204 (45.6)	0.020	0.888
Aggressive behavior	19 (33.9)	137 (30.6)	0.250	0.617
Self-injurious behavior	2 (3.6)	30 (6.7)	0.381	0.537
Suicidal behavior	4 (7.1)	52 (11.6)	1.014	0.314
Incoherence of thinking	24 (42.9)	202 (45.2)	0.109	0.741
Illogic of thinking	9 (16.1)	36 (8.1)	3.927	**0.048**
Symbolic thinking	1 (1.8)	1 (0.2)	-	0.210 [a]
Sophistic thinking	0 (0.0)	6 (1.3)	-	1.000 [a]
Experience of being revealed	3 (5.4)	21 (4.7)	0.000	1.000
Hallucination-delusion syndrome	11 (19.6)	127 (28.4)	1.922	0.166
Psychic automatism syndrome	1 (1.8)	3 (0.7)	-	0.377 [a]
Hypochondriac syndrome	3 (5.4)	8 (1.8)	1.528	0.216
Poverty of thought	25 (44.6)	276 (61.7)	6.057	**0.014**
Apathy	39 (69.6)	323 (72.3)	0.169	0.681
Abulia	35 (62.5)	316 (70.7)	1.584	0.208

[a] Fisher's exact test result

Table S7 Association between the deletion of PLA2G4A and schizophrenia clinical phenotypes in male

Symptoms	PLA2G4A CNV genotype Deletion [n (%)] (N=26)	Non-deletion [n (%)] (N=270)	χ^2	*P* value
Auditory hallucinations	16 (61.5)	176 (65.2)	0.138	0.710
Olfactory hallucinations	0 (0)	4 (1.5)	-	1.000 [a]
Visual hallucinations	1 (3.8)	5 (1.9)	-	0.427 [a]
Visceral hallucination	0 (0)	1 (0.4)	-	1.000 [a]
Psychosensory disturbance	1 (3.8)	3 (1.1)	-	0.309 [a]
Delusion of reference	20 (76.9)	195 (72.2)	0.264	0.608
Delusion of persecutory	20 (76.9)	195 (72.2)	0.264	0.608
Delusion of physical influence	0 (0)	17 (6.3)	0.768	0.381
Delusion of jealousy	6 (23.1)	31 (11.5)	1.952	0.162
Delusion of sin	1 (3.8)	4 (1.5)	-	0.371 [a]
Delusion of grandeur	1 (3.8)	21 (7.8)	0.115	0.735
Nihilistic delusion	0 (0)	2 (0.7)	-	1.000 [a]
Erotomanic delusion	0 (0)	2 (0.7)	-	1.000 [a]
Catatatonia	0 (0)	1 (0.4)	-	1.000 [a]
Bizarre behavior	10 (38.5)	111 (41.1)	0.069	0.793
Aggressive behavior	11 (42.3)	86 (31.9)	1.177	0.278
Self-injurious behavior	0 (0)	20 (7.4)	1.057	0.304
Suicidal behavior	0 (0)	31 (11.5)	2.222	0.136
Incoherence of thinking	12 (46.2)	121 (44.8)	0.017	0.896
Illogic of thinking	4 (15.4)	19 (7)	1.288	0.256
Symbolic thinking	1 (3.8)	1 (0.4)	-	0.168 [a]
Sophistic thinking	0 (0)	2 (0.7)	-	1.000 [a]
Experience of being revealed	1 (3.8)	5 (1.9)	-	0.427 [a]
Hallucination-delusion syndrome	2 (7.7)	61 (22.6)	3.143	0.076
Hypochondriac syndrome	2 (7.7)	6 (2.2)	-	0.150 [a]
Poverty of thought	8 (30.8)	155 (57.4)	6.801	**0.009**
Apathy	16 (61.5)	191 (70.7)	0.955	0.328
Abulia	16 (61.5)	189 (70)	0.798	0.372

[a] Fisher's exact test result

Table S8 Association between the deletion of PLA2G4A and schizophrenia clinical phenotypes in female

Symptoms	PLA2G4A CNV type		χ^2	*P* value
	Deletion [n (%)] (N=30)	Non-deletion [n (%)] (N=177)		
Auditory hallucinations	18 (60)	125 (70.6)	1.355	0.286
Visual hallucinations	0 (0)	5 (2.8)	-	1.000 [a]
Visceral hallucination	0 (0)	2 (1.1)	-	1.000 [a]
Psychosensory disturbance	0 (0)	4 (2.3)	-	1.000 [a]
Delusion of reference	26 (86.7)	144 (81.4)	0.493	0.483
Delusion of persecutory	21 (70)	119 (67.2)	0.090	0.764
Delusion of physical influence	4 (13.3)	23 (13)	0.000	1.000
Delusion of jealousy	5 (16.7)	31 (17.5)	0.013	0.910
Delusion of sin	0 (0)	1 (0.6)	-	1.000 [a]
Delusion of grandeur	0 (0)	6 (3.4)	-	0.596 [a]
Nihilistic delusion	0 (0)	1 (0.6)	-	1.000 [a]
Erotomanic delusion	4 (13.3)	5 (2.8)	4.519	**0.034**
Catatatonia	1 (3.3)	4 (2.3)	-	0.547 [a]
Bizarre behavior	15 (50)	93 (52.5)	0.066	0.797
Aggressive behavior	8 (26.7)	51 (28.8)	0.058	0.810
Self-injurious behavior	2 (6.7)	10 (5.6)	0.000	1.000
Suicidal behavior	4 (13.3)	21 (11.9)	0.000	1.000
Incoherence of thinking	12 (40)	81 (45.8)	0.344	0.557
Illogic of thinking	5 (16.7)	17 (9.6)	0.706	0.401
Sophistic thinking	0 (0)	4 (2.3)	-	1.000 [a]
Experience of being revealed	2 (6.7)	16 (9)	0.006	0.939
Hallucination-delusion syndrome	9 (30)	66 (37.3)	0.590	0.443
Psychic automatism syndrome	1 (3.3)	3 (1.7)	-	0.468 [a]
Hypochondriac syndrome	1 (3.3)	2 (1.1)	-	0.376 [a]
Poverty of thought	17 (56.7)	121 (68.4)	1.579	0.209
Apathy	23 (76.7)	132 (74.6)	0.060	0.807
Abulia	19 (63.3)	127 (71.8)	0.875	0.350

[a] Fisher's exact test result

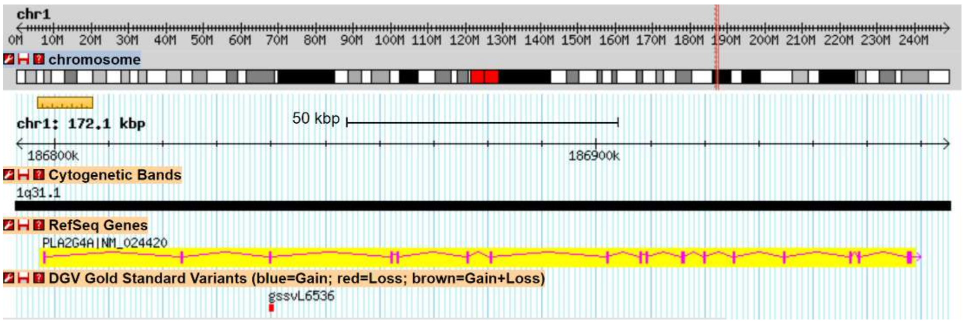

Figure S1 Schematic of the identified DGV Gold Standard CNV of PLA2G4A in Chr.1q31.1
